# Supplementary material for: The isomer distribution of C6H6 products from the propargyl radical gas-phase recombination investigated by threshold-photoelectron spectroscopy
Source: Commun Chem. 2024 Jul 12;7:156. doi: 10.1038/s42004-024-01239-7 (PMC11245511; doi:10.1038/s42004-024-01239-7)
Supplement: Supplementary file 2 — Supplementary Information [file 42004_2024_1239_MOESM2_ESM.pdf]

<u>**Electronic Supplementary Information**</u>

The isomer distribution of C_6H_6 products from the propargyl radical gas-phase recombination investigated by threshold-photoelectron spectroscopy

Helgi Rafn Hrodmarsson,*[a,b] Gustavo A. Garcia,[a] Lyna Bourehil,[a] Laurent Nahon,[a] Bérenger Gans,[c] Séverine Boyé-Péronne,[c] Jean-Claude Guillemin,[d] and Jean-Christophe Loison,*[e]

[a] *Synchrotron SOLEIL, L'Orme des Merisiers, St. Aubin, F-91192 Gif sur Yvette, France.*

[b] *Univ Paris Est Créteil and Université Paris Cité, CNRS, LISA UMR 7583, 94010 Créteil, France.*

[c] *Université Paris-Saclay, CNRS, Institut des Sciences Moléculaires d'Orsay, F-91405 Orsay, France*

[d] *Univ Rennes, École Nationale Supérieure de Chimie de Rennes, CNRS, ISCR – UMR6226, F-35000 Rennes, France*

[e] *Institut des Sciences Moléculaires, CNRS, Université de Bordeaux, F-33400 Talence, France*

Supplementary Note 1/ On the contribution of $C_3H_4F_2$ to the *m/z* 78 TPES

To verify the contribution of $C_3H_4F_2$ to the signal recorded for *m/z* 78, we first identified the species formed when atomic fluorine was present at high concentration, which favours multiple reactions with fluorine and thus potentially double addition. A characteristic mass spectrum of these conditions obtained in our previous study[1] of F + CH_3CCH scheme (with high F atomic concentration) and integrated over the photon energy 8.3-10.2 eV is shown in Fig. S1 lower panel (the mass spectrum for F << C_3H_4 is also shown for comparison in upper panel).

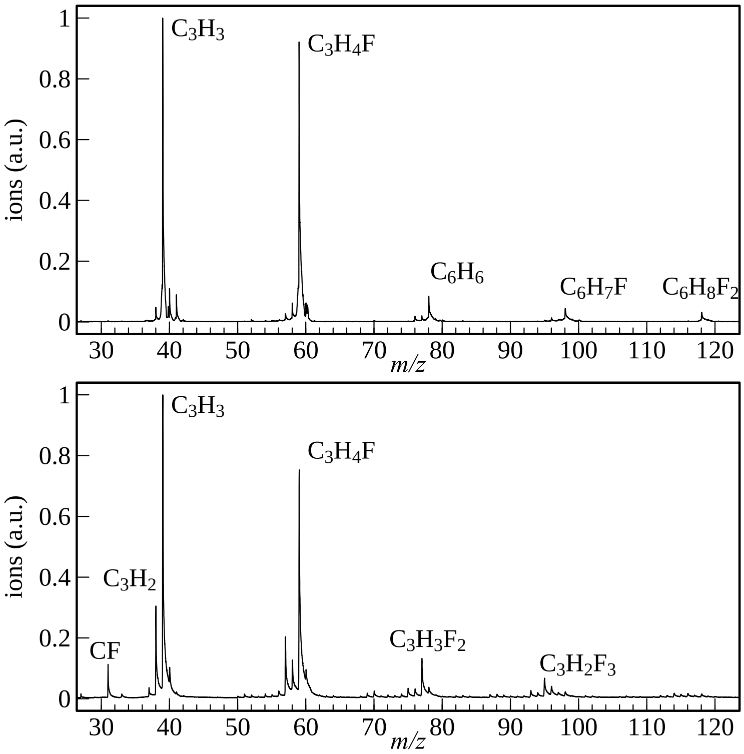

Figure S1: TOF mass spectra for the F + CH_3CCH system; Upper panel: integrated between 8.0 and 10.2 eV, with a low F concentration. Lower panel: integrated between 8.2 and 10.2 eV photon energy, with high F atom concentration.

With respect to the mass spectrum recorded with low F concentration (see upper panel of Fig. S1), we can see for high F atomic concentration, the strong increase of the multiple reactions with the fluorine atom: the double abstraction of the hydrogen atom to produce C_3H_2 and the multiple addition of the fluorine atoms. However, the strong decrease of *m/z* 78 (C_3H_6/ $C_3H_4F_2$) shows that $C_3H_4F_2$ is not, or very little, produced even with high abundances of fluorine. On the other hand, $C_3H_3F_2$ production (*m/z* 77) is favoured, corresponding to the reaction:

$C_3H_4F + F \rightarrow C_3H_3F_2 + H$

Actually, there are four isomers formed by the addition of atomic fluorine on methylacetylene. The first two are CH_3-C=CHF and CH_3-CF=CH (*m/z* 59):

$CH_3CCH + F \rightarrow CH_3\text{-C=CHF}$ $\quad \Delta_r H^0_{298}$= -196 kJ/mol

$CH_3CCH + F \rightarrow CH_3\text{-CF=CH}$ $\quad \Delta_r H^0_{298}$= -195 kJ/mol

CH_3-C=CHF can isomerize to the more stable moiety CH_2-CH=CHF *via* a transition state (TS) located -18 kJ/mol below the CH_3CCH + F entrance level resulting into:

$CH_3CCH + F \rightarrow CH_2\text{-CH=CHF}$ $\quad \Delta_r H^0_{298}$= -286 kJ/mol

The other isomer, CH_3-CF=CH, can isomerize to the more stable species CH_2-CF=CH_2 *via* a TS located -26 kJ/mol below the CH_3CCH + F entrance level resulting into:

$CH_3CCH + F \rightarrow CH_2\text{-CF=}CH_2$ $\quad \Delta_r H^0_{298}$= -296 kJ/mol

The TPES recorded for *m/z* 59 (corresponding to the products of the F addition on methylacetylene) is shown in Fig. S2 as black curve. Franck-Condon simulations for the different C_3H_3F isomers are displayed in panels a) to e) as green curves. We can see that the most abundant isomers are trans- and cis-CH_2-CH=CHF (panels a) and b), respectively) and CH_2-CF=CH_2 (panel c)). The Franck-Condon factors of the ionization of the CH_3-C=CHF (panel d)) and CH_3-CF=CH isomers (panel e)) to their singlet ground state is very unfavourable (the CH_3CFCH^+ ion does not appear to be stable and isomerizes into CH_3CHCF^+). On the other hand, their ionization toward their first excited state (with triplet spin multiplicity) have favourable Franck-Condon factors and are shown as green curves. Their (quasi) absence on the spectrum shows that the CH_3-C=CHF and CH_3-CF=CH isomers are not abundantly isomerizing into CH_2-CH=CHF and CH_2-CF=CH_2.

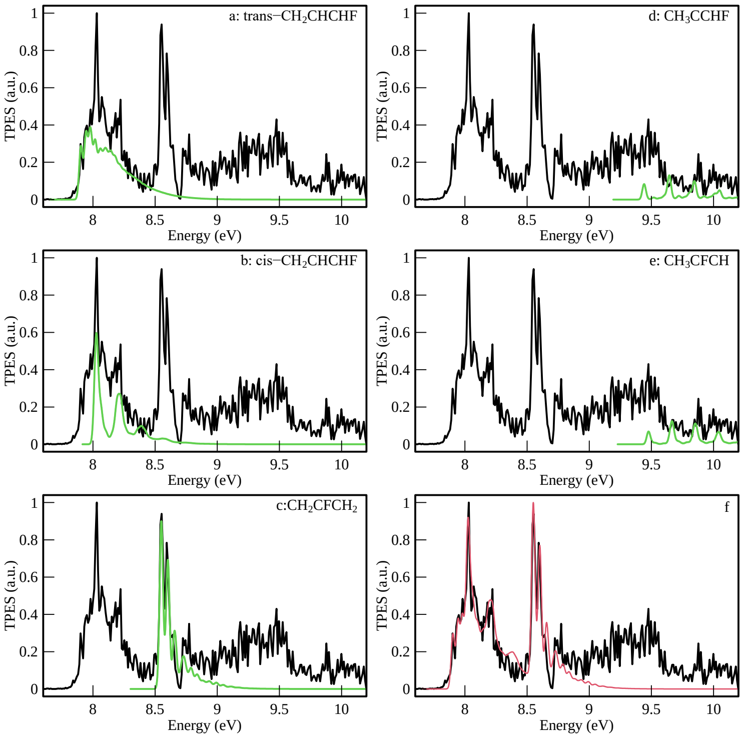

Figure S2: TPES recorded in the present work with a large F atom concentration for *m/z* 59 as black curve. Panels a) to e) display as green curves the Franck-Condon simulations performed for trans-CH_2-CH=CHF, cis-CH_2-CH=CHF, CH_2-CF=CH_2, CH_3-C=CHF (triplet ion state), CH_3-CF=CH (triplet ion state), respectively. Panel f) shows as red curve the composite spectrum resulting from addition of the simulations of panels a) to c) with Franck-Condon factors multiplied by 1, 0.57 and 0.68 respectively.

Table S3 summarizes the experimental adiabatic ionization energies measured in our work for three $C_3H_4F_2$ isomers.

Table S3: Experimental adiabatic ionization energy of trans-CH_2CHCHF, cis-CH_2CHCHF and CH_2CFCH_2 obtained in this work.

Species	IE_{ad} (eV)
trans-CH_2CHCHF	7.903 ± 0.005
cis-CH_2CHCHF	8.027 ± 0.005
CH_2CFCH_2	8.550 ± 0.005

With high abundances of fluorine, these four C_3H_4F isomers can react with an additional fluorine atom leading, for the addition products, to:

CH_3-C=CHF + F → CH_3-CF=CHF $\Delta_r H^0_{298}$= -507 kJ/mol

→ CH_2-CF=CHF + H $\Delta_r H^0_{298}$= -148 kJ/mol

CH_3-CF=CH + F → CH_3-CF=CHF $\Delta_r H^0_{298}$= -508 kJ/mol

→ CH_2-CF=CHF + H $\Delta_r H^0_{298}$= -149 kJ/mol

CH_2-CH=CHF + F → CH_2F-CH=CHF $\Delta_r H^0_{298}$= -407 kJ/mol

→ CHF-CH=CHF + H $\Delta_r H^0_{298}$= -61 kJ/mol

CH_2-CF=CH_2 + F → CH_2F-CF=CH_2 $\Delta_r H^0_{298}$= -404 kJ/mol

→ CH_2-CF=CHF + H $\Delta_r H^0_{298}$= -48 kJ/mol

The relatively high exothermicities of the dissociative pathways, associated with the absence of a barrier for these sigma C-H bond breaks, explains the near absence of $C_3H_4F_2$ (*m/z* 78) production and the production of $C_3H_3F_2$ (*m/z* 77) observed in the TOF mass spectrum of Fig.1 a) of the main article or in Fig. S1. The TPES of *m/z* 77 for the conditions where F is abundant is presented in Fig. S4 in black curve. Panels a), b) and c) of Fig. S4 show in green line the Franck-Condon simulations for the three $C_3H_3F_2$ isomers. Assuming similar photoionization cross sections, we conclude that the production of these three isomers occurs in similar proportion.

For completeness, panel d) shows the PES of the benzyl radical C_6H_5 (sharing the same *m/z* value) measured by Butcher *et al.*[2] From the comparison with our TPES, we conclude on the non-production of this dissociation product in our conditions (4 mbar of total pressure).

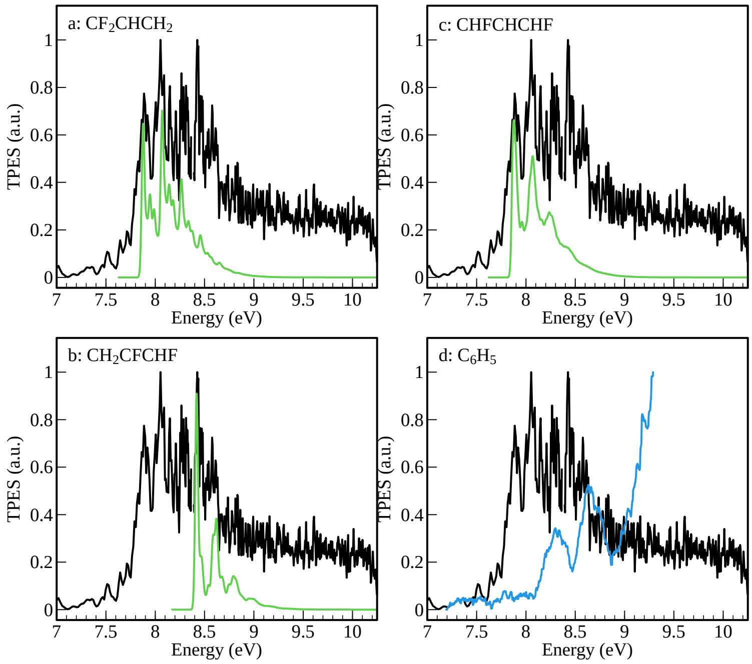

Figure S4: TPES recorded in the present work with a large F atom concentration for *m/z* 77 as black curve. Panels a) to c) display as green curves the Franck-Condon simulations performed for $C_3H_3F_2$ isomers, *i.e.* CF_2-CH=CH_2, CH_2-CF=CHF, and CHFCH=CHF, respectively. Panel d) shows the experimental C_6H_5 PES from Butcher *et al*.[2] as blue curve for comparison

In the TOF mass spectra of Fig. S1 upper panel (with [F] << [CH_3CCH]), the absence, or near absence, of $C_3H_4F_2$ in the signal contribution to the mass *m/z* 78 is also observed. The resolution of the mass spectrometer being around 1000, depending on the signal processing performed, we can see that the signal of the mass *m/z* 78 is mainly due to C_6H_6 (*m/z* 78.04695) and not to $C_3H_4F_2$ (*m/z* 78.02811) even if our resolution does not allow us to separate their masses significantly. It should be noted that for [F] << [CH_3CCH], the signal for the mass *m/z* = 77 is weak and that the absence of a structure for the C_6H_5 PES[2,3] makes it impossible to quantify its abundance, other than to say that it is low.

The non (or very small) contribution of $C_3H_4F_2$ to the *m/z* 78 mass is also confirmed by comparing the calculated photoionization spectra of the four isomers (shown in Fig. S5) of $C_3H_4F_2$ with the TPES of the *m/z* 78 mass for the [F] << [CH_3CCH] conditions.

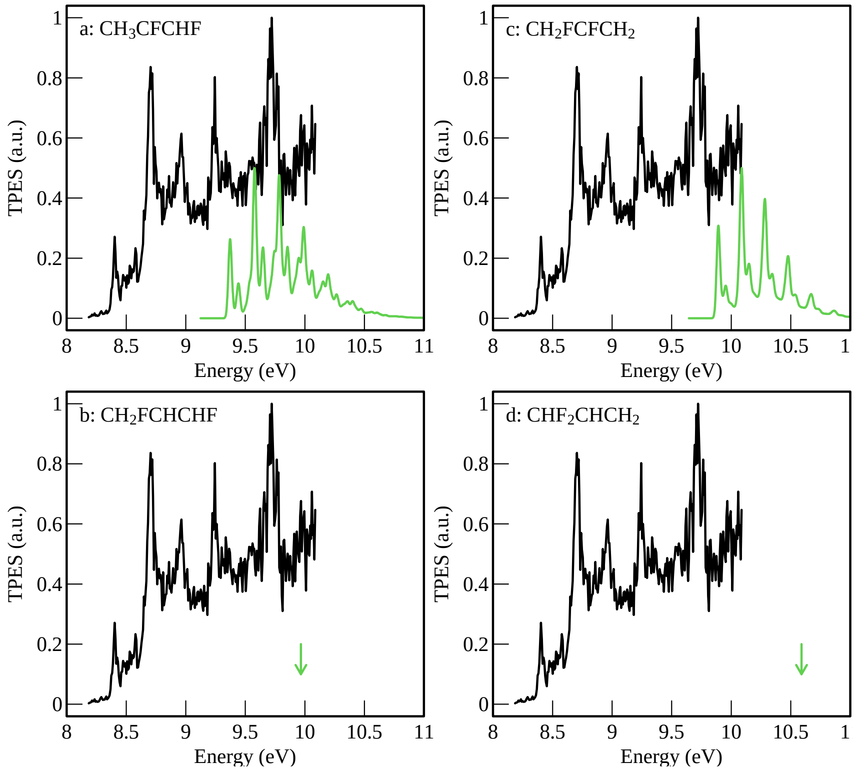

Figure S5: TPES recorded in the present work with a very low F atom concentration for *m/z* 78 as black curve. Panels a) and c) display as green curves the Franck-Condon simulations performed for CH_3-CF=CHF and CH_2F-CF=CH_2. For CH_2FCHCHF and CHF_2CHCH_2 (panels b) and d)); there is a large change in geometry between the neutral and the cation preventing Gaussian to calculate FC factors. Thus, the calculated adiabatic IE is only indicated with a green arrow.

Supplementary Note 2/ Monte-Carlo simulations of the weighting factors for the reference spectra summed to reproduce the TPES at *m/z* 78 (corresponding to Fig. 6 of the main article)

In our Monte-Carlo approach, as a starting point we adjust initial weights manually to reproduce the mixture spectrum as well as possible. Then we vary each distribution by ± - 0.2 (imposing a positive or zero value for each ratio) and as the ratios are lower (or very close to 0.2) we explore virtually all possibilities. In our Monte-Carlo approach, we consider all distributions with the same probability (uniform distribution), the only criterion for considering a set of ratios to be valid is that the average of the absolute difference between the experimental and new summed spectrum is smaller than 9.2% up to 9.9 eV (the numerical criterion value (9.2%) being not critical in the 9–10% range). It is worth noting that even if we use a uniform distribution for each ratio, rejecting distributions that do not meet the chosen criterion induces quasi-Gaussian distributions for branching ratios that reproduce the experimental spectrum. In Fig. S6 we represent the branching ratio distribution for each isomer.

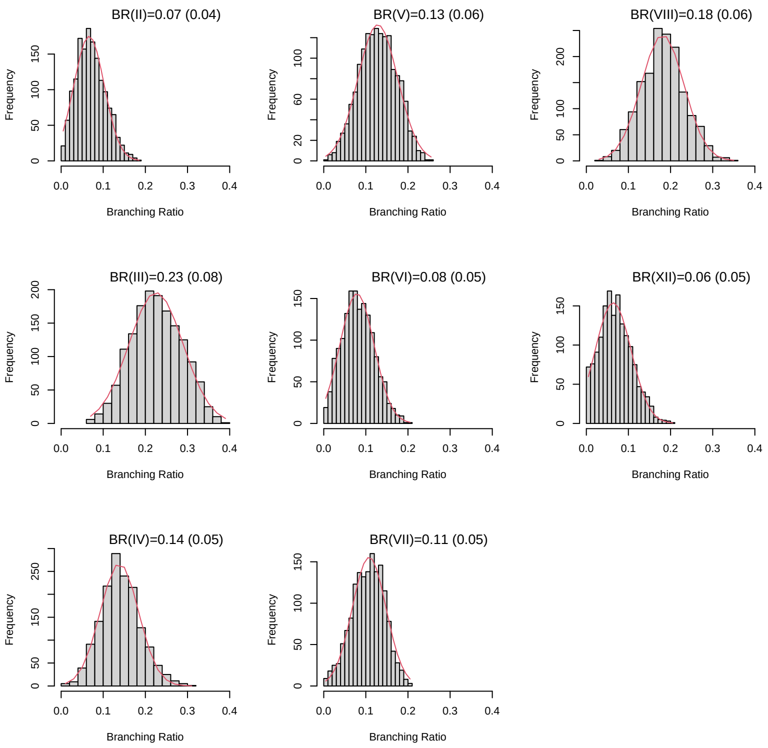

Figure S6: The simulated weighting factors and their statistical distributions during the Monte-Carlo approach to reproduce the experimental *m/z* 78 spectra (see Fig. 6 of the main article). The BR (branching ratio) values are given for each isomer.

In Fig. S7, we compare our experimental TPES with the weighted sum of all contributing reference spectra with the branching ratios determined in the present work (see Table 1 of the main text), as well as with the branching ratios from Miller and Klippenstein[4] (blue curve of Fig. S7). The agreement is significantly poorer when using the branching ratios from the Miller and Klippenstein study.[4] Deviations consist particularly in an underestimation of the bands at 8.7 eV (due to 13HD5Y), at 9.25 eV (due to benzene) and an overestimation of the bands at 9.0 and 9.18 eV (due to 2E13BD). This comparison enhances also the importance of 12HD5Y for reproducing the spectrum above 9.5 eV.

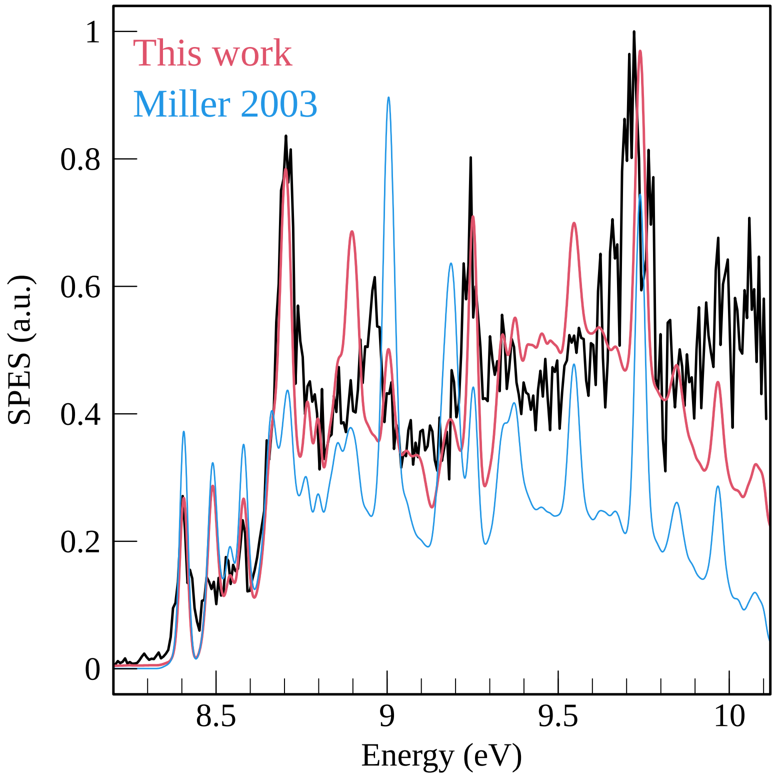

Figure S7: Experimental TPES of *m/z* = 78 in black, and comparison with the weighted sum of all contributing reference spectra with the branching ratios determined in the present work and reported in Table 1 (red curve), and with the branching ratios taken from Miller and Klippenstein[4] (blue curve).

Supplementary References

1. G. A. Garcia, B. Gans, J. Kruger, F. Holzmeier, A. Roder, A. Lopes, C. Fittschen, C. Alcaraz, J.-C. Loison, *Phys. Chem. Chem. Phys.*, 2018, **20**, 8707-8718.
2. V. Butcher, M. L. Costa, J. M. Dyke, A. R. Ellis, A. Morris, *Chem. Phys.*, 1987, **115**, 261.
3. J. Hrušák, D. Schröder, S. Iwata, *J. Chem. Phys.*, 1997, **106**, 7541-7549.
4. J. A. Miller, S. J. Klippenstein, *J. Phys. Chem. A.*, 2003, **107** (39), 7783-7799.
